# Some Obstetric Points

**Published:** 1890-12

**Authors:** George H. Rohé

**Affiliations:** Professor of Obstetrics, College of Physicians and Surgeons, Baltimore


### SOME OBSTETRIC POINTS.

By GEORGE H. ROHÉ, M. D.

Professor of Obstetrics, College of Physicians and Surgeons, Baltimore.

[*Baltimore Medical and Surgical Journal.*]

ANTISEPSIS means cleanliness. Septic fever after delivery means neglect of cleanliness.

THERE is no "milk fever." What has been called by that name is neither more nor less than fever from septic absorption.

DURING the past three years, 377 women were delivered in the Maryland Maternité. Not one of these died of any septic disease.

THE treatment of puerperal peritonitis by abdominal incision, flushing and drainage, is gaining ground. The opium treatment is being abandoned for saline purgation.

AT the Maternité, Credé's method of preventing ophthalmia neonatorum by applying a one per cent. solution of silver nitrate to the conjunctiva immediately after birth, is used with success.

THE old-fashioned "monthly nurse" must go. The clean, neat, intelligent and trained aseptic obstetric nurse is coming into fashion. Let us help her along in her good work.

CESAREAN section and craniotomy: They do not exclude each other but each has its rationally indicated field of usefulness. There are, properly speaking, no operations of election in obstetrics; the indicated operation is the one that must be done.

A DISINFECTANT solution for the hands should contain 1:1000 of mercuric chloride. This is to be used *after* the hands and arms have been scrubbed with hot water and soap, using a nail brush vigorously for several minutes.

A VAGINAL mercuric chloride douche may be of the strength of 1:4000; for intra-uterine use it should not be stronger than 1:5000, injected through Gardner's intra-uterine, soft-rubber catheter.

